# Acceptability of a 250 mg levofloxacin formulation in children receiving TB preventive treatment

**DOI:** 10.5588/ijtldopen.24.0569

**Published:** 2025-03-12

**Authors:** S.E. Purchase, J. Brigden, J.A. Seddon, N.A. Martinson, L. Fairlie, S. Staples, A. Poswa, T. Duong, H.S. Schaaf, A.C. Hesseling

**Affiliations:** ^1^ Desmond Tutu TB Centre, Department of Paediatrics and Child Health, Faculty of Medicine and Health Sciences, Stellenbosch University, Tygerberg, South Africa;; ^2^ Institute of Clinical Trials and Methodology, MRC Clinical Trials Unit at University College London, London, UK;; ^3^ Department of Infectious Disease, Imperial College London, London, UK;; ^4^ Perinatal HIV Research Unit, University of the Witwatersrand, Johannesburg, South Africa;; ^5^ Johns Hopkins University Center for TB Research, Baltimore, MD, USA;; ^6^ Wits RHI, Faculty of Health Sciences, University of the Witwatersrand, Johannesburg, South Africa;; ^7^ THINK, Durban, South Africa;; ^8^ Isango Lethemba TB Research Unit, Wits Health Consortium, University of the Witwatersrand, Johannesburg, South Africa.

**Keywords:** multidrug-resistant tuberculosis, paediatrics, prevention

## Abstract

**BACKGROUND:**

Recent evidence indicates that levofloxacin (LFX) is effective in preventing TB in individuals exposed to multidrug-resistant TB (MDR-TB). Despite the need for pediatric formulations, the 250 mg adult LFX formulation is affordable and widely used for TB treatment and prevention in children.

**METHODS:**

TB-CHAMP (Tuberculosis Child Multidrug-resistant Preventive Therapy ISRCTN92634082) was a trial of MDR-TB preventive treatment, comparing levofloxacin to placebo in children with MDR-TB exposure. Acceptability questionnaires were administered to caregivers at six timepoints during the 24-week treatment phase. Likert scales were used to grade 6 domains of acceptability, and a composite acceptability (CA) outcome was generated. Factors associated with acceptability were assessed using modified Poisson regression models to estimate risk ratios (RRs).

**RESULTS:**

Overall, 922 children were randomised, 453 to LFX and 469 to placebo. By Week 8, 25.1% of children on LFX had poor CA versus 6.2% receiving placebo (Weeks 0–24: RR 3.43, 95% CI 2.69–4.37). Acceptability in the LFX arm improved from 36.8% poor CA at baseline to 12.9% at Week 24. Only 11.7% of children swallowing tablets whole/halved had poor CA outcomes at Week 8, compared to 34.4% swallowing crushed/softened tablets.

**CONCLUSION:**

LFX 250 mg tablets have reasonable acceptability and could be used as an alternative to dispersible formulations, especially in children able to swallow tablets.

Multidrug-resistant TB (MDR-TB), caused by *Mycobacterium tuberculosis* (Mtb) resistant to isoniazid and rifampicin, is threatening global TB control.^1^ An estimated 2 million children are currently infected with MDR-Mtb globally, with roughly 30,000 developing MDR-TB disease each year.^2^ Modelling studies suggest that without contact management and MDR-TB preventive treatment (MDR-TPT),^3^ the 2035 End TB Strategy targets^4^ will not be met.

Evidence from recent randomised controlled trials indicates that levofloxacin (LFX) is effective in preventing TB in children exposed to MDR-TB in the household.^5,6^ Guidelines regarding MDR-TB prevention strategies have recently been updated by the WHO, and 6 months of daily LFX is now recommended as TPT in contacts exposed to MDR-TB.^7^ LFX is thus a key component of MDR-TB treatment and prevention strategies in children.^8,9^ Although dispersible and palatable formulations designed for children are a priority, LFX 250 mg formulations are inexpensive and widely used globally in TB treatment and prevention.

Acceptability, defined as ‘the overall ability and willingness of the patient to use, and its caregiver to administer, the medicine as intended’, may substantially affect a person’s experience of treatment and adherence.^10,11^ No drug or regimen is efficacious unless it can be taken by the intended target population as prescribed. Acceptability is determined by multiple domains (including usability, receptivity and integration),^12^ but taste and ease of preparation/administration are key factors impacting acceptability in children. No data is currently available on the acceptability of LFX in children measured during long-term treatment.

TB-CHAMP was a TB prevention trial comparing 6 months of daily LFX with a taste-matched placebo. We collected acceptability data from all participants at multiple time points. Here, we explore the acceptability of the LFX 250 mg formulation and taste-matched placebo over time in children. We also assess the impact of acceptability on adherence.

## METHODS

### Trial design, setting and population

TB-CHAMP (Tuberculosis Child Multidrug-resistant Preventive Therapy ISRCTN92634082) was a multi-site, cluster-randomised, double-blind, placebo-controlled trial investigating the efficacy and safety of 24 weeks of LFX for the prevention of TB in child household contacts of adults with infectious MDR-TB. TB-CHAMP was conducted at five sites in South Africa between 2017 and 2023.^13^ Adults were identified following a diagnosis of confirmed pulmonary MDR-TB and recruited if there was at least one child aged <5 years living in the same household. Children were recruited if exposure to the MDR-TB index patient had been substantial in the preceding 6 months and randomised to either LFX or placebo by household. Under protocol version 3.0 (September 2021), children 5–17 years with a positive IGRA or living with HIV, were also included. Acceptability questionnaires were administered to caregivers and older children at six timepoints during the 24-week treatment phase (Supplementary Figure S1).

### Study drug and administration

Study medications were manufactured by Macleods Pharmaceuticals, Mumbai, India, as LFX or matched LFX-placebo 250 mg functionally scored, uncoated tablets, prescribed using weight bands, and dosed at 15–20 mg/kg (maximum 750 mg) daily for 24 weeks (Supplementary Figure S2). Initial dosing was at the study site and observed by study staff; subsequent doses were given by caregivers at home. Various options for dosing were discussed with the caregivers, including a recommendation to mix study drugs with small amounts of food or milk as needed to support adherence. Caregivers completed a daily treatment card to track pill-swallowing. Pill counts took place at each visit. Poor adherence was defined as participants having taken <80% of prescribed study treatment doses.

### Data collection

Acceptability questionnaires were administered at baseline and Weeks 4, 8, 12, 16 and 24 under v1.0 of the protocol and at Weeks 4,8 and 24 from v2.0 (13 May 2019). Questionnaires were administered to caregivers of all participants who had taken any study drug since the last visit. The opinions of older children were also solicited if they were willing. The acceptability questionnaires consisted of six items soliciting ranked responses from caregivers regarding acceptability, 14 questions soliciting categorical responses regarding methods of study drug administration, and two questions asking whether the caregiver needed to force/coerce the child to take the study drug. A 5-point Likert scale was used to grade 6 domains of acceptability (taste, amount, preparation, softening, administration, tablet size).

### Statistical analysis

The analysis included all randomised children who took at least one dose of the study drug and had a questionnaire administered at least once. One child was randomised to LFX but was incorrectly prescribed a placebo – this participant was analysed according to the treatment taken. Binary outcomes were generated from the 5-point Likert scale – responses in the two most negative categories (e.g. ‘Dislike’ and ‘Dislike very much’) were considered poor acceptability. A composite acceptability (CA) outcome was pre-defined as having poor acceptability in any of the six domains.

Acceptability for the individual domains and the overall composite outcome were compared between LFX and placebo arms over time. Modified Poisson regression was used to estimate the risk ratio.^14^ Multilevel models accounted for within-participant correlation of responses across different follow-up visits, as well as for household clustering. The associations between poor acceptability and study site, age (treated as a time-dependent covariate and defined a priori as <1, 1 to <3, 3 to <5, and ≥5 years), gender and clinical characteristics (HIV exposure status, comorbidities) were assessed. Analyses were adjusted for the covariates chosen as a priori: treatment arm, study site, age group, and week of study visit.

Drug administration methods were described by arm and over time. The association between administration and age was assessed. The impact of the administration method on acceptability was assessed among children receiving LFX only. The relationship between acceptability and adherence was assessed within the LFX arm only by comparing the proportion of participants with poor adherence overall in those with versus without poor acceptability at Week 4.

No adjustments were made for multiple statistical tests. Statistical analyses were performed using Stata v16.0 or later (Stata Statistical Software: Release 16-18.; StataCorp LLC College Station, TX, USA: Stata, 2023.).

### Ethical considerations

The trial was approved by the Health Research Ethics Committee of Stellenbosch University, Stellenbosch (M16/02/009) and University of the Witwatersrand, Johannesburg, South Africa (160409), the South African Health Products Regulatory Agency, Pretoria (20160128) and the South African Department of Health, Pretoria, South Africa (DOH-27-0117-5309) and was registered in the ISRCTN registry (ISRCTN92634082). Informed consent was provided by all participants’ parents or legal guardians, and assent was provided by children aged ≥7 years. Data were stored using unique anonymised study-patient identifiers to maintain confidentiality.

## RESULTS

### Child characteristics

922 children were enrolled and randomised on TB-CHAMP, 453 to the LFX and 469 to the placebo arms. One child did not take any doses of the study drug, and another child’s caregiver did not complete any acceptability questionnaires - both children were in the placebo arm. One child was randomised to the LFX arm but incorrectly prescribed a placebo – this child’s data has been analysed according to the drug received. Thus, in this sub-study, data from 920 participants were analysed: 452 in the LFX and 468 in the placebo group.

Baseline characteristics of randomised children were similar between treatment arms (Table 1). Participants had a median age of 2.8 years (interquartile range (IQR), 1.4 to 4.2 years), with 90% aged <5 years. Overall, 51% were female, 2% were living with HIV, and 34% were HIV-exposed but uninfected.

**Table 1.** Baseline characteristics of child participants in the TB-CHAMP TB prevention trial.

	Levofloxacin *n* (%)	Placebo *n* (%)	Total *n* (%)
Total children randomised	452 (100)	468 (100)	920 (100)
Female sex	240 (53)	226 (48)	466 (51)
Age, years				
Median	3.0	2.6	2.8
IQR	1.4–4.3	1.3–4.1	1.4–4.2
Range	0.1–17.9	0.1–17.4	0.1–17.9
HIV status				
Positive*	10 (2)	9 (2)	19 (2)
HIV-exposed, not infected	153 (34)	160 (34)	313 (34)
HIV-unexposed	287 (64)	297 (64)	584 (64)
Missing, *n*	2	2	4
BCG vaccination status				
No	28 (6)	25 (5)	53 (6)
Yes	422 (94)	441 (95)	863 (94)
Missing, *n*	2	2	4
Previously received any TB treatment, yes^†^	10 (2)	8 (2)	18 (2)
Currently on TB preventive treatment, yes	9 (2)	6 (1)	15 (2)
Weight-for-age *Z* score, *n*^‡^	452	468	920
Median	–0.4	–0.4	–0.4
IQR	–1.2 to 0.3	–1.2 to 0.4	–1.2 to 0.3
Range	–7.2 to 4.2	–6.2 to 5.1	–7.2 to 5.1
Height-for-age *Z* score, *n*^‡^	452	468	920
Median	–0.9	–0.9	–0.9
IQR	–1.6 to –0.1	–1.8 to –0.2	–1.7 to –0.2
Range	–6.8 to 8.8	–6.1 to 3.3	–6.8 to 8.8
Weight-for-length *Z* score, *n*^‡^	400	426	826
Median	0.1	0.3	0.3
IQR	–0.6 to 1.0	–0.4 to 1.1	–0.5 to 1.0
Range	–7.9 to 4.8	–4.8 to 5.7	–7.9 to 5.7
Missing, *n*	52	42	94
Site 1	222 (49)	230 (49)	452 (49)
2	92 (20)	80 (17)	172 (19)
3	116 (26)	141 (30)	257 (28)
4	3 (1)	4 (1)	7 (1)
5	19 (4)	13 (3)	32 (3)

* 14 (74%) of HIV-positive children were on antiretroviral treatment, 8 in the levofloxacin arm and 6 in the placebo arm.

† All treated for drug-susceptible TB, apart from 1 child with unknown relevant information.

‡ Standardised to the WHO reference, and for weight-for-age, to UK reference for age >10 years (since WHO weight-for-age growth charts are only available for age ≤10 years). Of note, WHO weight-for-length growth charts are only available for height range 65–120 cm; therefore, weight-for-length *Z*-score is considered missing for children with height outside this range.

TB-CHAMP = Tuberculosis Child Multidrug-resistant Preventive Therapy ISRCTN92634082; BCG = bacille Calmette-Guérin; IQR = interquartile range

### Acceptability of levofloxacin versus levofloxacin-placebo

The Figure summarises the acceptability outcomes across all six domains (See Supplementary Table S1 for details). Acceptability improved over time in both arms and for all outcomes: in the LFX arm, the CA outcome improved from 36.8% reporting poor acceptability at treatment initiation to 12.9% reporting poor acceptability at Week 24 (risk ratio [RR] 0.38, 95% confidence interval [CI] 0.27–0.53) (see Supplementary Table S1). There was a significant difference between treatment arms for all six acceptability outcomes, with caregivers experiencing the LFX as less palatable and more difficult to prepare and administer than the placebo. In adjusted analysis, poor composite acceptability was associated with younger age but not with gender, study site, HIV exposure status, or the presence of comorbidities (Table 2).

**Figure.**
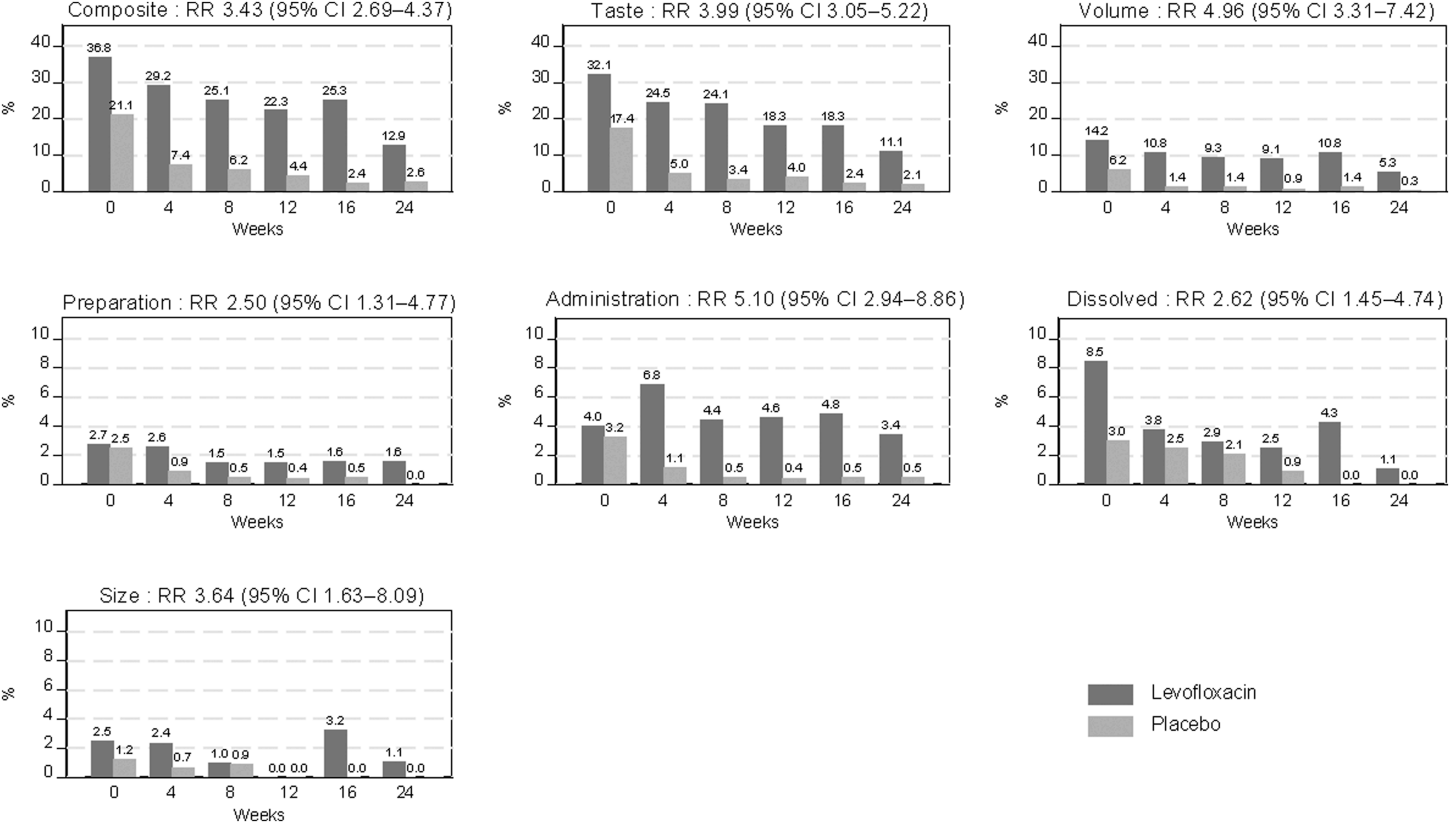
Proportion of children receiving levofloxacin or matched placebo with poor acceptability scores by week of study visit (see Supplementary Figure S1 for questions asked). RR = relative risk (comparing children receiving levofloxacin versus placebo); CI = confidence interval.

**Table 2.** Association of poor composite acceptability outcome with demographic and clinical characteristics by study treatment arm and over time (including Week 0–24 data).

Factors	Levofloxacin		Placebo		Overall		*p* value for between treatment group heterogeneity test
	RR (95% CI)	*p* value	RR (95% CI)	*p* value	RR (95% CI)	*p* value	
Sex							
Male	1		1		1		
Female	1.18 (0.96–1.45)	0.119	0.89 (0.66–1.21)	0.472	1.09 (0.92–1.29)	0.334	0.140
Age, years							
<1	1		1		1		
1 to <3	1.12 (0.88–1.43)		1.40 (0.90–2.18)		1.19 (0.96–1.47)		
3 to <5	0.80 (0.61–1.04)		0.90 (0.57–1.42)		0.82 (0.65–1.03)		
≥5	0.47 (0.30–0.73)	<0.001	0.98 (0.45–2.14)	0.046	0.56 (0.38–0.85)	<0.001	0.355
Site							
Site 1	1		1		1		
Site 2	1.26 (0.94–1.69)		0.81 (0.45–1.48)		1.09 (0.84–1.43)		
Site 3	0.74 (0.53–1.04)		0.64 (0.35–1.19)		0.70 (0.51–0.97)		
Site 5	1.33 (0.85–2.07)	0.024	0.56 (0.12–2.55)	0.455	1.07 (0.66–1.73)	0.088	0.460
HIV exposure							
No	1		1		1		
Yes	0.97 (0.78–1.21)	0.771	0.70 (0.46–1.09)	0.113	0.88 (0.73–1.07)	0.210	0.202
Comorbidities							
No	1		1		1		
Yes	1.23 (0.86–1.77)	0.252	1.22 (0.72–2.08)	0.453	1.23 (0.91–1.66)	0.179	0.980

Site with highest number of enrolments used as reference; Site 4 was removed from the analysis when comparing sites since it had a very small number of children (*n* = 7).

RR = risk ratio; CI = confidence interval.

### Administration methods

Table 3 summarises administration methods used by caregivers between arms and over time. At Week 12, 29.9% of children had swallowed LFX whole in the past month, 7.1% had swallowed halved, 42.1% of caregivers had given crushed LFX tablets and 45.7% had softened the tablets in water before administering them to the child. The composite outcome for children swallowing whole/halved increased from 38.8% at Week 0 in the LFX arm to 45.5% at Week 24.

**Table 3.** Administration of study treatment over time in children receiving LFX 250 mg or placebo for TB prevention.

		Week 0	Week 4	Week 8	Week 12	Week 16	Week 24
Tablet swallowed whole with liquid	LFX	134/402 (33.3)	133/425 (31.3)	145/410 (35.4)	59/197 (29.9)	68/186 (36.6)	158/378 (41.8)
	Placebo	134/403 (33.3)	170/444 (38.3)	196/435 (45.1)	93/227 (41.0)	100/211 (47.4)	191/386 (49.5)
Tablet swallowed halved with liquid	LFX	59/402 (14.7)	55/425 (12.9)	50/410 (12.2)	14/197 (7.1)	23/186 (12.4)	51/377 (13.5)
	Placebo	63/403 (15.6)	65/444 (14.6)	55/435 (12.6)	30/227 (13.2)	33/211 (15.6)	52/386 (13.5)
Tablet crushed	LFX	126/401 (31.4)	167/424 (39.4)	162/408 (39.7)	82/195 (42.1)	73/185 (39.5)	138/375 (36.8)
	Placebo	97/400 (24.2)	104/444 (23.4)	94/430 (21.9)	60/223 (26.9)	46/209 (22.0)	80/384 (20.8)
Tablet softened (‘dissolved’) in a liquid solution	LFX	225/402 (56.0)	210/424 (49.5)	178/410 (43.4)	90/197 (45.7)	73/186 (39.2)	143/378 (37.8)
	Placebo	228/403 (56.6)	219/444 (49.3)	199/435 (45.7)	103/226 (45.6)	84/210 (40.0)	166/384 (43.2)
Child was restrained or forced	LFX	72/401 (18.0)	81/424 (19.1)	71/410 (17.3)	33/197 (16.8)	26/186 (14.0)	47/378 (12.4)
	Placebo	64/403 (15.9)	15/443 (3.4)	13/435 (3.0)	2/227 (0.9)	3/211 (1.4)	6/386 (1.6)
Child was bribed or coerced	LFX	39/401 (9.7)	65/424 (15.3)	58/410 (14.1)	25/197 (12.7)	22/186 (11.8)	40/378 (10.6)
	Placebo	45/403 (11.2)	20/443 (4.5)	16/435 (3.7)	11/227 (4.8)	9/211 (4.3)	6/386 (1.6)
Composite outcome for tablet swallowed whole or halved with liquid*	LFX	156/402 (38.8)	156/425 (36.7)	162/410 (39.5)	68/197 (34.5)	77/186 (41.4)	172/378 (45.5)
	Placebo	158/403 (39.2)	193/444 (43.5)	212/435 (48.7)	105/227 (46.3)	109/211 (51.7)	210/386 (54.4)
Composite outcome for if child was restrained/forced or bribed/coerced*	LFX	83/401 (20.7)	100/424 (23.6)	94/410 (22.9)	43/197 (21.8)	39/186 (21.0)	62/378 (16.4)
	Placebo	83/403 (20.6)	27/443 (6.1)	20/435 (4.6)	11/227 (4.8)	11/211 (5.2)	11/386 (2.8)

* These were pre-defined.

LFX = levofloxacin.

At Week 12, 16.8% of children in the LFX arm had to be restrained/forced, and 12.7% of children had to be bribed/coerced to swallow LFX. Including Week 4–24 data, children in the LFX arm were 5 times as likely to be forced/bribed to take treatment than in the placebo arm (RR 5.49, 95% CI 3.98–7.56). Children aged 1 to <3 years were >7 times more likely to be forced/bribed to take treatment than children aged >5 years (≥5 years: RR 0.22, 95% CI 0.10–0.50; 1 to <3 years: RR 1.56, 95% CI 1.11–2.18; Supplementary Table S3).

As expected, the proportion of participants who could swallow the study drug whole or halved increased with age at randomisation. Overall, 65.6% of children aged 3–<5 years given LFX were able to swallow tablets whole/halved at some point during the trial (Supplementary Table S4). In children across all ages, only 11.7% of those swallowing whole/halved tablets had a poor CA score at Week 8, versus 34.4% of those swallowing tablets crushed/softened (Supplementary Table S5).

### Impact of acceptability on treatment adherence

Adherence was good in both arms, with 87% of children in the LFX arm and 86% in the placebo arm taking ≥80% of prescribed doses. Among children taking LFX, there did not appear to be a relationship between poor adherence and the taste of the medication, difficulty with administration, or the CA outcome. However, children with caregivers who reported finding it challenging to prepare the medication at Week 4 were 3.39 times (95% CI 1.53–7.52) more likely to have poor overall adherence than those who did not find preparation difficult (Table 4).

**Table 4.** Acceptability and adherence at Week 4 on study in children receiving 250 mg levofloxacin.

Acceptability outcome		Number of participants	Proportion of doses missed Median [IQR]	Proportion of participants discontinuing treatment early *n* (%)	Proportion of participants with poor adherence (took <80% of doses)	Risk ratio (95% CI)	*p* value
Child/adolescent disliked very much/disliked the taste of medication	No	321	4 [1–10]	39 (12.1)	35 (10.9)	1	
	Yes	104	4.5 [2–12]	18 (17.3)	16 (15.4)	1.47 (0.81–2.65)	0.201
Caregiver found it very difficult/difficult to prepare of study medication	No	414	4 [2–11]	53 (12.8)	47 (11.4)	1	
	Yes	11	4 [1–60]	4 (36.4)	4 (36.4)	3.39 (1.53–7.52)	0.003
Caregiver found it very difficult/difficult to administer the doses	No	396	4 [1–10]	52 (13.1)	46 (11.6)	1	
	Yes	29	10.5 [3.5–18]	5 (17.2)	5 (17.2)	1.29 (0.53–3.14)	0.568
Composite acceptability outcome, based on responses to Q1–6 on Form 14	No	301	4 [1–10]	36 (12.0)	32 (10.6)	1	
	Yes	124	4 [2–12]	21 (16.9)	19 (15.3)	1.48 (0.84–2.61)	0.177

IQR = interquartile range; CI = confidence interval.

## DISCUSSION

In this first trial of MDR-TB prevention in children, the LFX 250 mg formulation had reasonable acceptability, with 75% of participants reporting good acceptability by Week 8. Reported acceptability measures improved over time in children in both arms. Poor acceptability was associated with being younger – this is likely due to the need to manipulate tablets for younger children unable to swallow whole/halved. LFX was less well-tolerated than placebo across all ages.

Caregivers used a variety of methods to administer LFX. Although the formulation is not technically dispersible, many caregivers softened the tablets in water before administering them to younger children. Others crushed the tablets and administered them with food or water. Crushing, softening and mixing tablets with food or liquids is a common practice among caregivers of children taking TB medications,^15^ but this may negatively impact palatability and acceptability, especially in medications with a bitter taste.^16,17^ Many children 3–<5 years of age learnt to swallow the 250 mg tablets whole/halved. Acceptability scores were better in children able to swallow tablets without prior manipulation. There was no clear relationship between acceptability and treatment adherence, although the children of caregivers who struggled to prepare the medication were less likely to take >80% of prescribed doses.

LFX is known to have an extremely bitter taste that is difficult to mask in liquid and dispersible formulations. The development of a taste-matched placebo for TB-CHAMP involved adding an embittering agent. Our results show, however, that taste-matching was not perfect, as there was a clear difference in reported acceptability between study arms. Taste-matching bitter substances is challenging, and inadequate matching in clinical trials is probably more common than expected.^18^ Imperfect taste-matching is unlikely to have introduced bias in this double-blind placebo-controlled trial due to household clustering and the fact that many children taking placebo also had poor acceptability. Analyses in the acceptability sub-study also accounted for the treatment group when assessing the effects of other factors. Poorer acceptability scores for LFX across all domains may imply that poor palatability influences acceptability in other domains, as parameters such as size and volume should be identical between placebo and LFX arms.

The only association between acceptability and adherence was for caregivers who found it difficult to prepare medication. In this study, children took medication in a clinical trial scenario, which may have improved adherence and influenced associations. However, it is becoming increasingly clear that multiple factors, including support systems, health knowledge, access to resources and trust in patient-provider relationships, play a very important role in determining adherence, not only drug or formulation factors.^19^

Poor palatability and refusal of medication are common barriers to the implementation of isoniazid preventive treatment in children.^20^ The use of fluoroquinolones in children has historically been restricted due to potential concerns regarding damage to soft tissues of weight-bearing joints.^21^ Therefore, there are limited data on the acceptability of LFX in children. LFX has been used for many years, however, to treat children with MDR-TB disease. In one study, LFX for MDR-TB treatment given as crushed LFX tablets had poor acceptability among South African children.^16^ Data from the pharmacokinetic lead-in study in TB-CHAMP showed that most caregivers preferred a new child-friendly dispersible LFX 100 mg formulation over the 250 mg formulation received in routine care.^22^ This was confirmed by Wademan and colleagues, who directly compared the acceptability of 100 mg LFX dispersible and non-dispersible 250 mg tablet formulations in children receiving MDR-TPT.^23^ Nested in-depth qualitative research, including in-depth interviews with caregivers and children in TB-CHAMP, found that young children who required tablet manipulation to swallow disliked the bitter taste of the 250 mg formulation; however, older children who were able to swallow tablets whole found the tablet more acceptable.^24^ Our study corroborates and extends these previous findings, showing that in a large cohort, young children especially disliked the taste of the LFX 250 mg tablets but quickly learnt to swallow tablets whole and adapted to the taste over time.

We collected data from many children at multiple time points in a randomised placebo-controlled trial across five diverse sites in South Africa. This enabled a comparison of acceptability between treatment arms over time and an analysis of correlations between method of administration and acceptability, acceptability and adherence. Acceptability questionnaires were administered to caregivers by study personnel, and caregiver answers may have been impacted by their desire to please study staff. However, given the double-blind nature of the trial, answers would not have been likely affected by the treatment arm. Our analysis included mainly younger children and thus cannot adequately represent older children and adolescents. We acknowledge the importance of evaluating young children directly in future and are currently conducting a study investigating the acceptability of a drug-resistant TB regimen (with LFX), successfully including children as young as 3 years. The impact of poor acceptability on adherence may have been mitigated by the clinical trial environment and may not truly represent the impact of poor acceptability under programmatic conditions. Therefore, the results cannot necessarily be fully generalised to other population groups in other settings. The scope of questions in the questionnaire could have been more expansive and could have explored broader aspects of acceptability outside of those relating to palatability and administration only.

In conclusion, the LFX 250 mg formulation has reasonable acceptability and could be used as an alternative to dispersible 100 mg child-friendly formulations for TB prevention and treatment, where paediatric formulations are not yet available. However, high proportions of younger children who are less able to swallow tablets whole or halved found the formulation unacceptable. In light of the recent 2024 WHO TPT guidelines,^7^ which recommend 6 months of LFX as TPT for high-risk contacts of MDR-TB, it is vital for TB programmes to budget for and acquire, child-friendly formulations of LFX expediently, and make these available for younger children.

## Supplementary Material


