# Supplementary material for: Acceptability of a 250 mg levofloxacin formulation in children receiving TB preventive treatment
Source: IJTLD Open. 2025 Mar 12;2(3):129–36. doi: 10.5588/ijtldopen.24.0569 (PMC11906031; doi:10.5588/ijtldopen.24.0569)
Supplement: Supplementary file 1 [file ijtldopen24-0569_supplementarydata1.docx]

**Acceptability of a 250 mg levofloxacin formulation in children receiving TB preventive treatment**

**Supplementary Data**

**Figure S1: TB-CHAMP palatability and acceptability questionnaire**


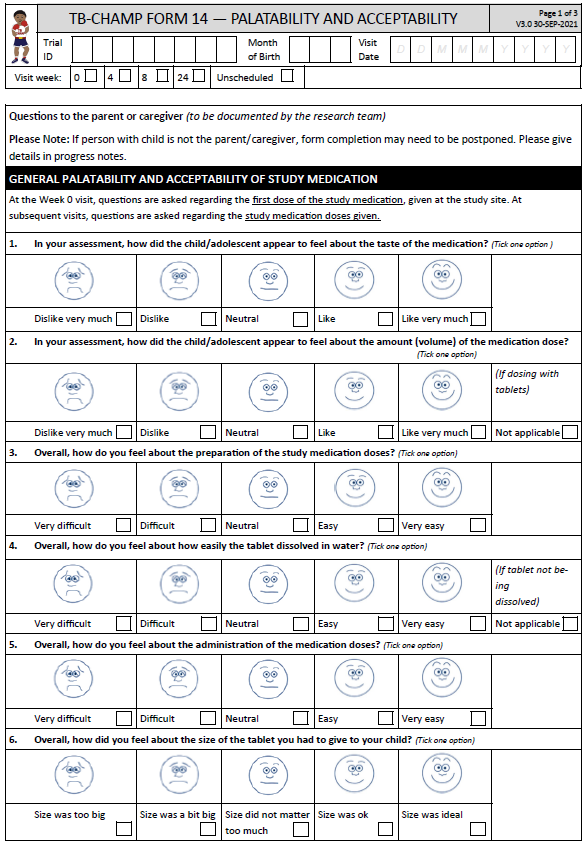


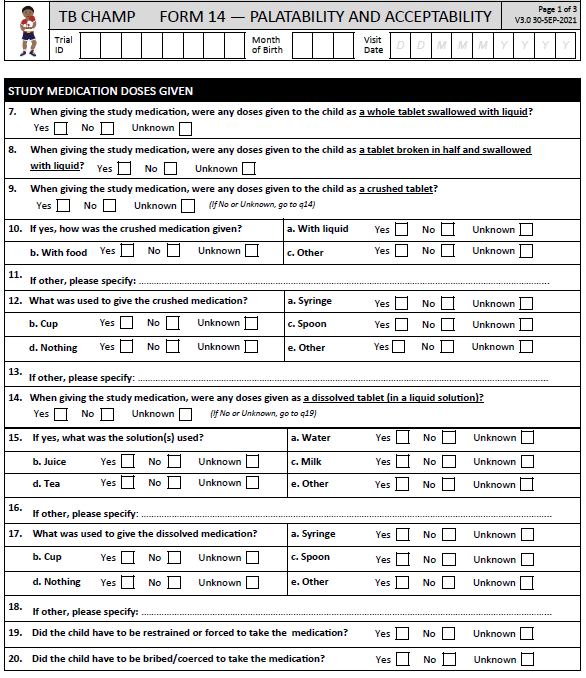


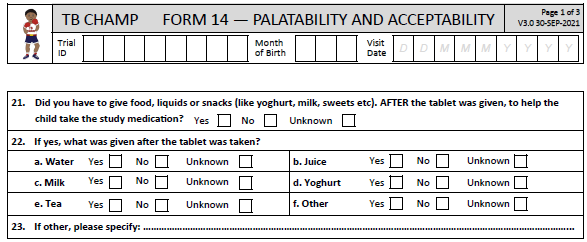


**Figure S2: Levofloxacin 250 mg formulation and matched placebo - Macleods Pharmaceuticals (Mumbai, India)**


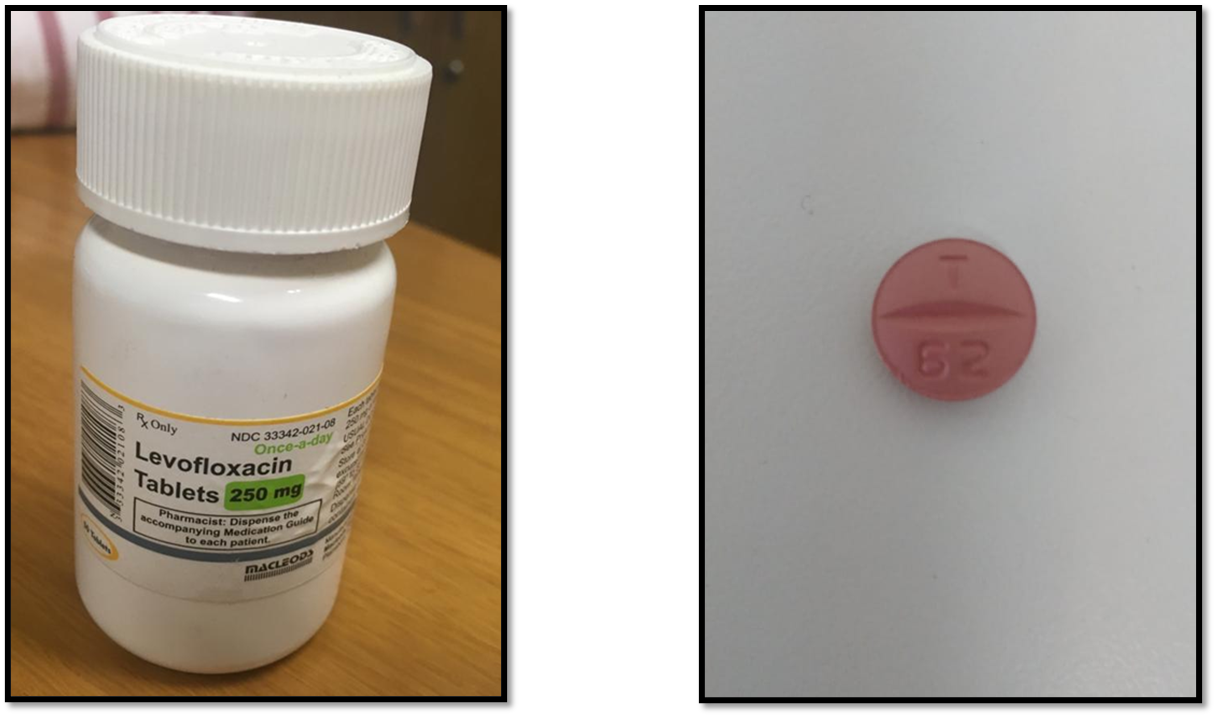


**Table S1: Summary of acceptability of study treatment over time in levofloxacin and placebo arms**

|  | | **Week 0** | **Week 4** | **Week 8** | **Week 12*** | **Week 16*** | **Week 24** | **Risk ratio (95% CI) comparing levofloxacin vs placebo Week 0-24** |
| --- | --- | --- | --- | --- | --- | --- | --- | --- |
| Overall number of participants with questionnaire completed |  | 805 | 869 | 845 | 424 | 397 | 765 |  |
| Child/adolescent disliked very much/disliked the **taste** of medication | Levofloxacin | 129/402 (32.1%) | 104/425 (24.5%) | 99/410 (24.1%) | 36/197 (18.3%) | 34/186 (18.3%) | 42/379 (11.1%) | 3.99 (3.05,5.22) |
|  | Placebo | 70/403 (17.4%) | 22/444 (5.0%) | 15/435 (3.4%) | 9/227 (4.0%) | 5/211 (2.4%) | 8/386 (2.1%) |  |
| Child/adolescent disliked very much/disliked the **amount** of medication dose | Levofloxacin | 57/402 (14.2%) | 46/425 (10.8%) | 38/410 (9.3%) | 18/197 (9.1%) | 20/186 (10.8%) | 20/379 (5.3%) | 4.96 (3.31,7.42 |
|  | Placebo | 25/403 (6.2%) | 6/444 (1.4%) | 6/435 (1.4%) | 2/227 (0.9%) | 3/211 (1.4%) | 1/386 (0.3%) |  |
| Caregiver found it very difficult/difficult to **prepare** the study medication | Levofloxacin | 11/402 (2.7%) | 11/425 (2.6%) | 6/410 (1.5%) | 3/197 (1.5%) | 3/186 (1.6%) | 6/379 (1.6%) | 2.50 (1.31,4.77) |
|  | Placebo | 10/403 (2.5%) | 4/444 (0.9%) | 2/435 (0.5%) | 1/227 (0.4%) | 1/211 (0.5%) | 0/386 (0.0%) |  |
| Caregiver found it very difficult/difficult to **soften** tablet in water | Levofloxacin | 34/402 (8.5%) | 16/425 (3.8%) | 12/410 (2.9%) | 5/197 (2.5%) | 8/186 (4.3%) | 4/379 (1.1%) | 2.62 (1.45,4.74) |
|  | Placebo | 12/403 (3.0%) | 11/444 (2.5%) | 9/435 (2.1%) | 2/227 (0.9%) | 0/211 (0.0%) | 0/386 (0.0%) |  |
| Caregiver found it very difficult/difficult to **administer** the doses | Levofloxacin | 16/402 (4.0%) | 29/425 (6.8%) | 18/410 (4.4%) | 9/197 (4.6%) | 9/186 (4.8%) | 13/379 (3.4%) | 5.10 (2.94,8.86) |
|  | Placebo | 13/403 (3.2%) | 5/444 (1.1%) | 2/435 (0.5%) | 1/227 (0.4%) | 1/211 (0.5%) | 2/386 (0.5%) |  |
| Caregiver felt the **size** of the tablet was too big/a bit big | Levofloxacin | 10/402 (2.5%) | 10/425 (2.4%) | 4/410 (1.0%) | 0/197 (0.0%) | 6/186 (3.2%) | 4/379 (1.1%) | 3.64 (1.63,8.09) |
|  | Placebo | 5/403 (1.2%) | 3/444 (0.7%) | 4/435 (0.9%) | 0/227 (0.0%) | 0/211 (0.0%) | 0/386 (0.0%) |  |
| **Composite poor acceptability outcome**, based on responses to Q1-6 on Form 14 | Levofloxacin | 148/402 (36.8%) | 124/425 (29.2%) | 103/410 (25.1%) | 44/197 (22.3%) | 47/186 (25.3%) | 49/379 (12.9%) | 3.43 (2.69,4.37) |
|  | Placebo | 85/403 (21.1%) | 33/444 (7.4%) | 27/435 (6.2%) | 10/227 (4.4%) | 5/211 (2.4%) | 10/386 (2.6%) |  |

* No questionnaires administered at Week 12 and 16 timepoints after Protocol Version 1.0, hence smaller numbers

### **Table S2: Summary of poor composite acceptability of study treatment over time**

| **Time point** | **n disliked composite/N (%)** | | **Risk ratio (95% CI) for change over time** | | |
| --- | --- | --- | --- | --- | --- |
|  | **LVX** | **Placebo** | **LVX** | **Placebo** | **Overall** |
|  |  |  |  |  |  |
| **Week 0** | 148/402 (36.8%) | 85/403 (21.1%) | 1 | 1 | 1 |
| **Week 4** | 124/425 (29.2%) | 33/444 (7.4%) | 0.84 (0.69,1.02) | 0.38 (0.26,0.57) | 0.67 (0.56,0.80) |
| **Week 8** | 103/410 (25.1%) | 27/435 (6.2%) | 0.72 (0.59,0.89) | 0.32 (0.20,0.52) | 0.58 (0.47,0.70) |
| **Week 12** | 44/197 (22.3%) | 10/227 (4.4%) | 0.60 (0.45,0.82) | 0.24 (0.12,0.45) | 0.47 (0.35,0.62) |
| **Week 16** | 47/186 (25.3%) | 5/211 (2.4%) | 0.69 (0.50,0.94) | 0.13 (0.05,0.34) | 0.48 (0.36,0.65) |
| **Week 24** | 49/379 (12.9%) | 10/386 (2.6%) | 0.38 (0.27,0.53) | 0.14 (0.07,0.28) | 0.29 (0.21,0.39) |
|  |  |  | **P<0.001** | **P<0.001** | **P<0.001** |

### **Table S3: Summary of administration of study treatment methods by age group in levofloxacin and placebo arms**

|  | | | **Including week 0-24 data** | | **Including week 4-24 data** | |
| --- | --- | --- | --- | --- | --- | --- |
|  | |  | **RR (95% CI)** | **P** | **RR (95% CI)** | **P** |
| **1.Child was restrained or forced** | | | | | |  |
|  | Treatment Arm | Placebo | 1 |  | 1 |  |
|  |  | Levofloxacin | 3.86 (2.92,5.12) | P<0.001 | 8.06 (5.42,11.97) | P<0.001 |
|  | Age | <1yrs | 1 |  | 1 |  |
|  |  | 1 to <3 yrs | 1.35 (1.02,1.77) |  | 1.36 (0.96,1.92) |  |
|  |  | 3 to <5 yrs | 0.53 (0.37,0.75) |  | 0.52 (0.33,0.81) |  |
|  |  | >=5yrs | 0.15 (0.08,0.31) | P<0.001 | 0.15 (0.07,0.33) | P<0.001 |
| **2.Child was bribed or coerced** | | | | | |  |
|  | Treatment Arm | Placebo | 1 |  | 1 |  |
|  |  | Levofloxacin | 3.05 (2.17,4.28) | P<0.001 | 4.83 (3.22,7.26) | P<0.001 |
|  | Age | <1yrs | 1 |  | 1 |  |
|  |  | 1 to <3 yrs | 2.97 (1.88,4.71) |  | 3.65 (2.04,6.52) |  |
|  |  | 3 to <5 yrs | 2.19 (1.37,3.52) |  | 2.64 (1.45,4.80) |  |
|  |  | >=5yrs | 0.69 (0.28,1.73) | P<0.001 | 0.78 (0.26,2.35) | P<0.001 |
| **3. Child was either restrained/forced or bribed/coerced** | | | | | |  |
|  | Treatment Arm | Placebo | 1 |  | 1 |  |
|  |  | Levofloxacin | 3.09 (2.44,3.92) | P<0.001 | 5.49 (3.98,7.56) | P<0.001 |
|  | Age | <1yrs | 1 |  | 1 |  |
|  |  | 1 to <3 yrs | 1.51 (1.16,1.98) |  | 1.56 (1.11,2.18) |  |
|  |  | 3 to <5 yrs | 0.81 (0.59,1.10) |  | 0.83 (0.57,1.22) |  |
|  |  | >=5yrs | 0.23 (0.11,0.45) | P<0.001 | 0.22 (0.10,0.50) | P<0.001 |

### **Table S4: Number of participants able to swallow study drug whole/halved**

|  | | **At Week 0** | **Any time during Week 4-24** |
| --- | --- | --- | --- |
| Overall |  | 314/805 (39.0%) | 516/895 (57.7%) |
| Levofloxacin group |  | 156/402 (38.8%) | 240/439 (54.7%) |
| Placebo group |  | 158/403 (39.2%) | 276/456 (60.5%) |
| **For levofloxacin group only** |  |  |  |
| Age group (years) | <1 year | 7/73 (9.6%) | 16/73 (21.9%) |
|  | 1 to <3 years | 22/122 (18.0%) | 48/165 (29.1%) |
|  | 3 to <5 years | 84/159 (52.8%) | 128/195 (65.6%) |
|  | ≥5 years | 43/48 (89.6%) | 80/90 (88.9%) |
| Gender | Male | 73/185 (39.5%) | 113/207 (54.6%) |
|  | Female | 83/217 (38.2%) | 127/232 (54.7%) |

### **Table S5: Association between administration method and acceptability in levofloxacin arm**

| **Acceptability outcome** |  | **Week 0** | **Week 4** | **Week 8** | **Week 12** | **Week 16** | **Week 24** |
| --- | --- | --- | --- | --- | --- | --- | --- |
| Number (%) of participants who disliked taste of medication | Tablets swallowed | 29/156 (18.6%) | 20/156 (12.8%) | 18/162 (11.1%) | 6/68 (8.8%) | 11/77 (14.3%) | 10/172 (5.8%) |
|  | Crushed/softened | 103/262 (39.3%) | 90/285 (31.6%) | 87/262 (33.2%) | 32/134 (23.9%) | 24/113 (21.2%) | 33/220 (15.0%) |
| Number (%) of participants with poor overall acceptability (composite outcome) | Tablets swallowed | 36/156 (23.1%) | 23/156 (14.7%) | 19/162 (11.7%) | 6/68 (8.8%) | 14/77 (18.2%) | 12/172 (7.0%) |
|  | Crushed/softened | 120/262 (45.8%) | 108/285 (37.9%) | 90/262 (34.4%) | 40/134 (29.9%) | 34/113 (30.1%) | 39/220 (17.7%) |
